# Ruptured aneurysm at the fenestration of the middle cerebral artery detected by magnetic resonance angiography in a patient with systemic lupus erythematosus and renal failure: a case report

**DOI:** 10.1186/1752-1947-8-30

**Published:** 2014-01-27

**Authors:** Sadaharu Tabuchi, Hiroki Yoshioka

**Affiliations:** 1 Department of Neurosurgery, Tottori Prefectural Central Hospital, 730 Ezu, Tottori 680-0901, Japan

**Keywords:** Arterial fenestration, Cerebral aneurysm, Magnetic resonance angiography, Renal failure, Subarachnoid hemorrhage, Systemic lupus erythematosus

## Abstract

**Introduction:**

A cerebral aneurysm arising at the fenestration of the middle cerebral artery is extremely rare, with one report describing subarachnoid hemorrhage due to this type of lesion. There have been no reports of this type of lesion occurring in a patient with systemic lupus erythematosus.

**Case presentation:**

A 47-year-old Japanese woman with 23 years’ history of systemic lupus erythematosus and chronic renal failure had sudden onset of subarachnoid hemorrhage. We avoided using contrast medium due to her chronic renal failure. Magnetic resonance angiography showed her ruptured aneurysm arising at the site of fenestration of her middle cerebral artery. Successful clipping, perioperative management avoiding the cerebral vasospasm, renal dialysis initiated after the acute phase and placement of a ventriculoperitoneal shunt were performed, and she was discharged home with no complications.

**Conclusions:**

This is the first report of ruptured aneurysm associated with middle cerebral artery fenestration in a patient with systemic lupus erythematosus as detected by magnetic resonance angiography. The presence and anatomical relationship of fenestration accompanied by aneurysm could be noninvasively and accurately evaluated preoperatively using three-dimensional time-of-flight magnetic resonance angiography with the volume rendering method in a case in which contrast medium was contraindicated.

## Introduction

The reported incidence of fenestration of the middle cerebral artery (MCA) ranges from 0.26 to 0.28% [1]. Although MCA fenestration is extremely rare, an aneurysm can arise at the proximal end of the fenestration. The magnetic resonance angiographic features of an aneurysm associated with MCA fenestration have rarely been reported. Subarachnoid hemorrhage (SAH) occurs in about 1% of patients with systemic lupus erythematosus (SLE) [2]. There are no previous reports of ruptured aneurysm at the MCA fenestration in a patient with SLE. The present report describes a case that was correctly diagnosed preoperatively by magnetic resonance angiography (MRA). This modality was used because the presence of underlying chronic renal failure was a contraindication to other imaging modalities and in order to avoid the initiation of dialysis during the acute phase of SAH due to the potential of cerebral vasospasm.

## Case presentation

**Figure 1:**
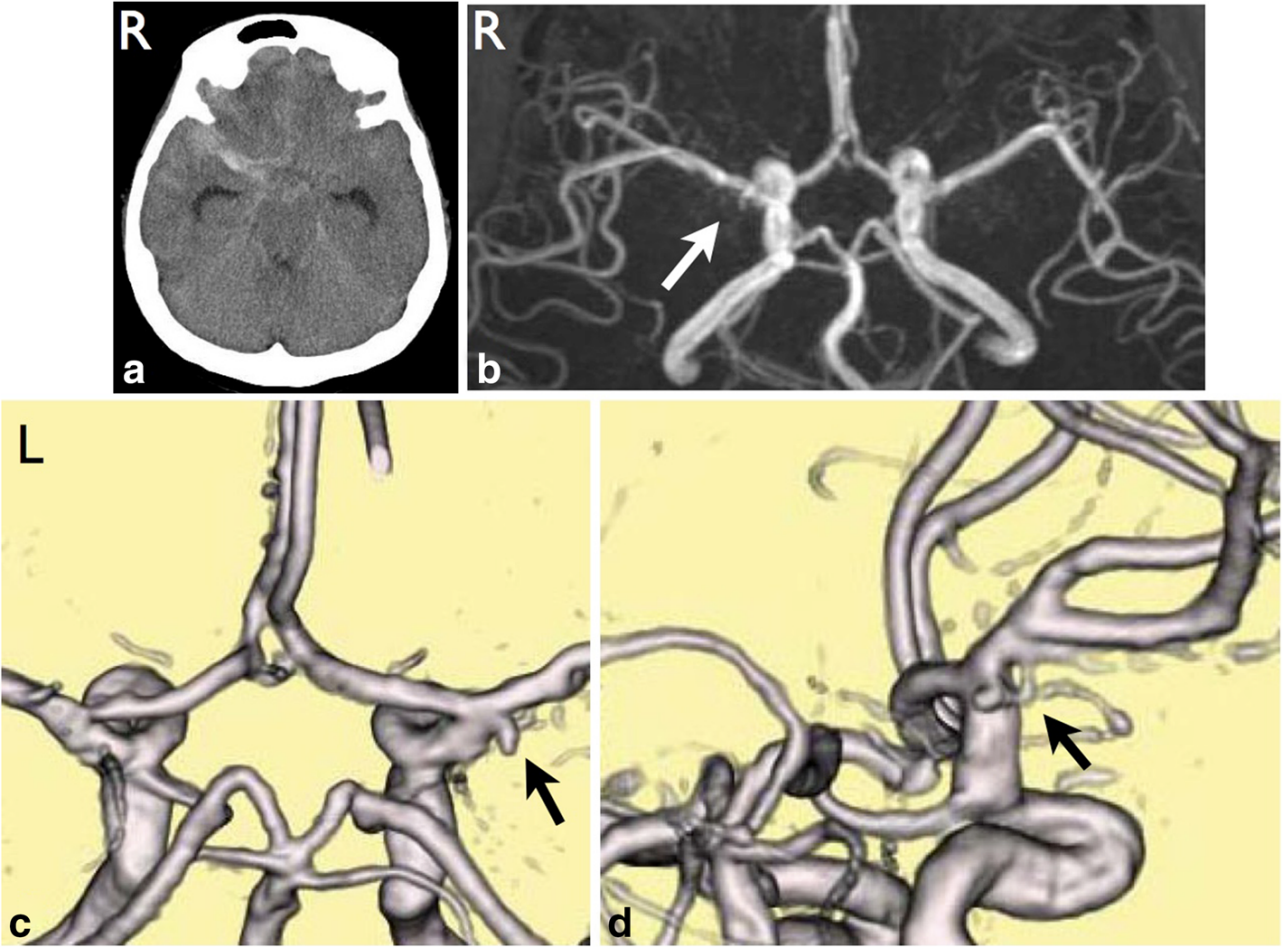
**Pre-operative images. (a)** Plain computed tomography shows subarachnoid hemorrhage in the right Sylvian fissure. **(b)** Maximum intensity projection image of magnetic resonance angiography suggests the presence of a ruptured aneurysm at the right proximal M1 portion (arrow). **(c)** Volume rendering image of magnetic resonance angiography shows the aneurysm arising at the fenestration of M1 (arrow). **(d)** Different angle of the volume rendering image shows the anatomical relationship between the fenestration and the aneurysm (arrow). R, Right side; L, Left side.

**Figure 2:**
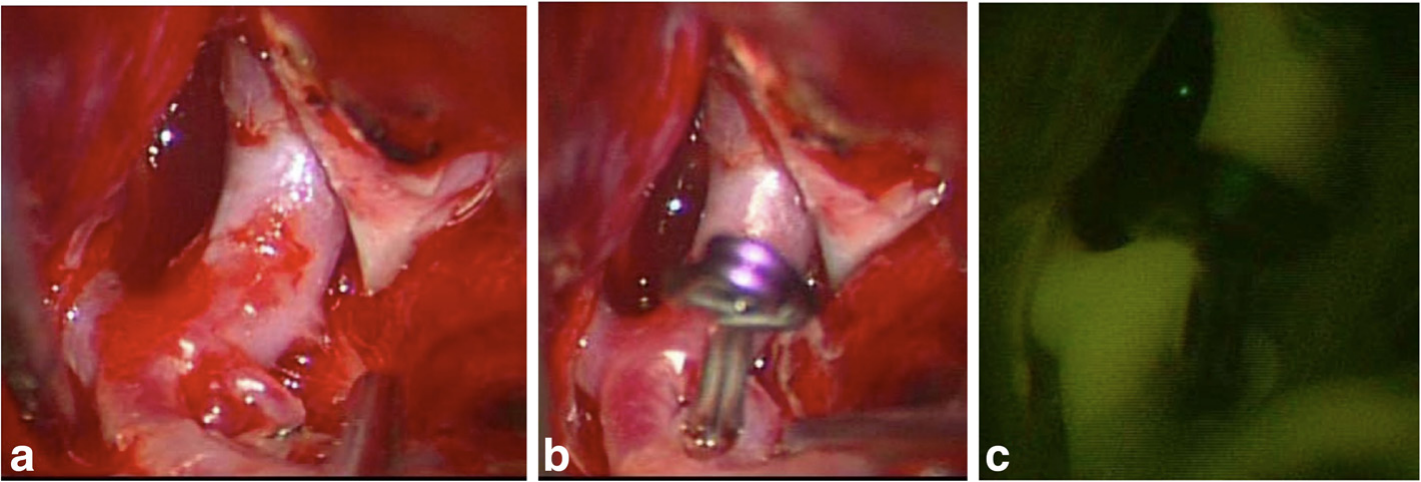
**Intra-operative images. (a)** Intraoperative view of the ruptured aneurysm arising at the proximal portion of the middle cerebral artery fenestration. **(b)** Intraoperative view after clipping of the aneurysm. **(c)** Intraoperative indocyanine green video angiography shows the middle cerebral artery fenestration and complete disappearance of the aneurysm.

A 47-year-old Japanese woman with 23 years’ history of SLE had sudden onset of severe headache and was referred to our hospital. She had been treated as an out-patient with a daily prednisolone dose of 7.5mg and cyclophosphamide of 50mg/day before the onset. Her glomerular filtration rate (GFR) was 5.6mL/minute /1.73m^2^ and the stage was classified as G5 (end stage) of chronic kidney disease (CKD) on admission. Laboratory examinations revealed hypocomplementemia (complement C3: 66mg/dL, C4: 14mg/dL). Her anti-double-stranded deoxyribonucleic acid (DNA) antibody (IgG) was 6.5IU/mL (normal range <20). Computed tomography (CT) demonstrated SAH in her right Sylvian fissure (Figure 1a). Considering that she had SLE and had developed chronic renal failure without renal dialysis, MRA was used to identify her ruptured aneurysm. MRA was performed using a 1.5T magnetic resonance system (Signa HDxt, GE, USA). Contrast material was not used. The volume rendering (VR) method with three-dimensional time-of-flight (TOF) MRA was performed (Figure 1c,d) in addition to the usual maximum intensity projection (MIP) image (Figure 1b). MRA revealed a fenestration at the M1 portion of her right MCA and a small saccular aneurysm at the proximal end of the fenestration (Figure 1c,d). This was consistent with a ruptured aneurysm. A left frontotemporal craniotomy was performed to clip the neck of her aneurysm on the same day. The fenestration and ruptured aneurysm were confirmed under surgical microscopy, and the aneurysm was successfully occluded (Figure 2a,b). Intraoperative indocyanine green videoangiography confirmed this result (Figure 2c). The present patient was carefully managed avoiding cerebral vasospasm and general complications during the perioperative period and renal dialysis was initiated after the acute phase and had a favorable outcome. Postoperatively, she developed normal pressure hydrocephalus, requiring placement of a ventriculoperitoneal shunt. She was ultimately discharged home in good condition.

## Discussion

Fenestration is a rare cerebrovascular variation. Fenestration of the MCA, which is present in 0.26 to 0.28% of patients, is much less frequent than fenestration of the vertebrobasilar system [1]. Most of the fenestrations reported previously have been located at the proximal portion of M1 [3,4]. It is rare to see aneurysms at the proximal end of the fenestration [5]. There has been only one reported case of ruptured aneurysm at this fenestration [6].

SLE is an autoimmune systemic inflammatory disease. Although SAH due to ruptured aneurysm has rarely been reported in patients with SLE, SAH in Japanese patients with SLE is more frequent than in patients from Western countries and can occur regardless of SLE disease activity [7]. The mortality rate associated with SAH is relatively high (38.6%) in Japanese patients with SLE [7].

This is the first report of SAH from a ruptured aneurysm associated with MCA fenestration in a patient with SLE. As this patient had developed severe chronic renal failure due to the diffuse lupus nephritis, typical imaging modalities were contraindicated, and MRA was utilized for diagnosis and preoperative planning. Digital subtraction angiography (DSA) and three-dimensional CT angiography are currently considered the modalities of choice for imaging and presurgical assessment of intracranial aneurysms. However, both methods require contrast medium, and DSA is a relatively invasive method and is associated with a risk of various complications. Some patients cannot undergo these imaging modalities due to renal failure (as in the present case) or an allergy to the contrast medium. CKD (GFR <60mL/minute/1.73m^2^) is a risk factor for contrast-induced nephropathy. CKD (GFR <30mL/minute/1.73m^2^) without dialysis is generally considered a contraindication for usage of contrast medium according to the Kidney Disease – Improving Global Outcomes 2012 clinical practice guideline for the evaluation and management of CKD.

MRA is considered a safe imaging technique because it is noninvasive, nonradioactive, technically simple, and does not require the administration of a contrast medium. Furthermore, this technique allows three-dimensional imaging of intracranial structural details. Recently, the utility of the VR method with the three-dimensional TOF-MRA has been described [8,9]. Although the MIP technique has been generally used, VR imaging yields better results in terms of spatial recognition. Indeed, the presence and anatomical relationship between fenestration and aneurysm was characterized using this technique. This imaging technique also facilitated surgical planning in this case.

The use of contrast medium and initiation of dialysis were avoided during the acute phase of SAH due to the high risk of cerebral vasospasm. As the prognosis of SLE patients associated with SAH can be poor, both diagnosis and therapy requires special attention in such conditions.

## Conclusion

This is the first report of a ruptured aneurysm associated with MCA fenestration in a patient with SLE in which MRA was used to correctly diagnose the condition preoperatively.

## Consent

Written informed consent was obtained from the patient for publication of this case report and accompanying images. A copy of the written consent is available for review by the Editor-in-Chief of this journal.

## Abbreviations

CKD: Chronic kidney disease; CT: Computed tomography; DSA: Digital subtraction angiography; GFR: Glomerular filtration rate; MCA: Middle cerebral artery; MIP: Maximum intensity projection; MRA: Magnetic resonance angiography; SAH: Subarachnoid hemorrhage; SLE: Systemic lupus erythematosus; TOF: Time-of-flight; VR: Volume rendering.

## Competing interests

The authors declare that they have no competing interests.

## Authors’ contributions

ST was the major contributor in writing the manuscript. HY reviewed the manuscript. Both authors read and approved the final manuscript.
